# Simple and reliable treatment for post-EMR artificial ulcer floor with snare cauterization for 10- to 20-mm colorectal polyps: a randomized prospective study (with video)

**DOI:** 10.1007/s00464-014-3983-y

**Published:** 2014-12-06

**Authors:** Hirohito Mori, Hideki Kobara, Noriko Nishiyama, Shintaro Fujihara, Tae Matsunaga, Maki Ayaki, Taiga Chiyo, Tsutomu Masaki

**Affiliations:** 1 Department of Gastroenterology and Neurology, Faculty of Medicine, Kagawa University, 1750-1 Ikenobe, Kita-gun, Miki-cho, Kagawa 761-0793 Japan; 2 Department of Gastroenterological Surgery, Ehime Rosai Hospital, 13-27 Minamikomatsubara, Niihama, Ehime 792-8550 Japan

**Keywords:** Endoscopic mucosal resection, Delayed bleeding prevention, Snare cauterization, Clip closure, Medical costs

## Abstract

**Background:**

Comparative studies on wound surface treatments after endoscopic mucosal resection (EMR) of 10- to 20-mm colorectal polyps have not been reported. We conducted a prospective trial of postoperative hemorrhage prevention measures after EMR of such polyps.

**Methods:**

Of 138 patients (397 polyps) who had undergone EMR, 62 patients (148 polyps) with 10- to 20-mm colorectal polyps were enrolled. Using the sealed envelope method, the subjects were randomly assigned to either a snare cauterization (75 polyps) or clip closure group (73 polyps). The primary assessment item was the wound surface treatment time (from immediately after polyp resection to wound surface treatment completion). The secondary assessment items were the incidence of delayed bleeding, perforation incidence 1–7 days after EMR, and difference in medical costs between the groups (*University Hospital Medical Information Network: No. 000013473*).

**Results:**

The time required for wound surface treatment completion was 3.26 ± 1.57 min in the snare cauterization group and 12.7 ± 2.92 min in the clip closure group, thus demonstrating a significant difference (*P* = 0.0001). Delayed bleeding was observed in two patients in the clip group, but was not observed in the snare cauterization group (*P* = 0.098). The clip group required the use of 720 clips that cost \523,410, US $5,163.50, or €3,665.5.

**Conclusions:**

After EMR of with 10- to 20-mm colorectal polyps, snare cauterization was superior to clip closure in terms of procedure time, and medical costs, and not inferior to clip closure in terms of the preventing effect of delayed bleeding.

**Electronic supplementary material:**

The online version of this article (doi:10.1007/s00464-014-3983-y) contains supplementary material, which is available to authorized users.

Wound clip closure after endoscopic mucosal resection (EMR) still entails many controversial problems in terms of prevention of delayed bleeding, such as remnant retention. Several reports stated that delayed bleeding occurred in approximately 0.98 % of cases 1–7 days after EMR of small polyps <10 mm (mean size, 7.8 mm); however, the use and nonuse of clip closure yielded no significant difference [1, 2].

On the contrary, other reports stated that clip closure resulted in a delayed bleeding incidence of approximately 1.4 % and thus, prevented delayed bleeding 1–7 days after endoscopic submucosal dissection (ESD) of polyps >20 mm [3]. These reports stated that in patients with polyps <10 mm, the incidence of delayed bleeding was 0.98 % even without clip closure, but those who underwent ESD of polyps >20 mm required clip closure to prevent delayed bleeding. Thus, reports have shown that the incidence of delayed bleeding increases from 0.98 % for tumors <10 mm after EMR to 1.4 % for tumors >20 mm after ESD. However, comparative trials of clip closure or other wound surface treatments for under 20-mm colorectal tumors after EMR have not yet been reported. Moreover, no evidence has been found regarding the optimal treatment of wounds incurred during EMR of colorectal tumors of this size. In addition, the question of how to reduce medical costs has also become an important issue.

Therefore, we compared the effectiveness of the following two methods of preventing delayed bleeding after EMR for under 20-mm colorectal tumors: the use of clips and the use of post-resection cautery of the wound surface using a snare tip. We have also examined the effects of these respective methods on medical costs.

## Patients and methods

### Patients and study protocol

After approval from the institutional review boards of the hospitals, colonoscopy was performed to 286 patients between March and August 2014, and 138 patients (397 polyps) were diagnosed with colorectal polyps at either Ehime Rosai Hospital or Kagawa University Hospital. We defined inclusion criteria as diameters between 10 and 20 mm as and exclusion criteria as diameters <10 and >20 mm. The endoscope featured a leading-end attachment with an inner diameter of 11 mm; therefore, polyps that adhered to the attachment were assessed to be >10 mm in diameter, whereas those requiring two or more attachments were assessed to be >20 mm in diameter.

Total of 249 polyps (76 patients) were excluded as following reasons: 231 polyps (70 patients) under 10 or over 20 mm were excluded. Ten polyps (4 patients) declined to participate in this trial. Eight polyps (2 patients) were excluded due to involving advanced colon cancer. Then, we selected as 62 patients (148 lesions) out of 397 polyps (138 patients) with 10- to 20-mm colorectal polyps.

In the next week conference after colonoscopy examination, we assigned numbers to polyps, and randomly allocated polyps with odd number as a snare cauterization group (*n* = 75) or polyps with even number as a clip closure group (*n* = 73) using the sealed envelope method. Regarding the ethical aspects of this study, all the patients were hospitalized for 3 days two nights to monitor for possible delayed bleeding after EMR.

Endoscopists who performed the EMRs were specialist members of the Japan Gastroenterological Endoscopy Society (Drs. T.T., N.I., and Dr. Y.H.). Before EMR, the endoscopists were instructed by Dr. H.M., as to which wound surface treatment to perform after EMR; they then performed EMR accordingly. The attending physician was a general internist with no involvement in the EMRs. The treatment of patients who were using anticoagulants was changed to heparin 4 days before EMR (maintained PT-INR 1.5), and heparin was then discontinued 4 h before EMR. Heparin was subsequently resumed 4 h after EMR, and anticoagulants were resumed the next day. For patients who were taking antiplatelet drugs and who had advanced cardiovascular disease, we consulted a cardiologist and requested adjustment of their medications and wash out period as follows: ①The oral administration of Aspirin 100 mg/day, 3 days before EMR ②The oral administration of Ticlopidine Hydrochloride 200 mg/day, 5 days before EMR ③The oral administration of Clopidogrel Sulfate 75 mg/day, 5 days before EMR. All antiplatelet drugs were resumed the next day.

Pretreatment of the colon consisted of ingestion of 2 L of a polyethylene glycol solution (Niflec, Ajinomoto Pharma Co., Tokyo, Japan).

**Fig. 1:**
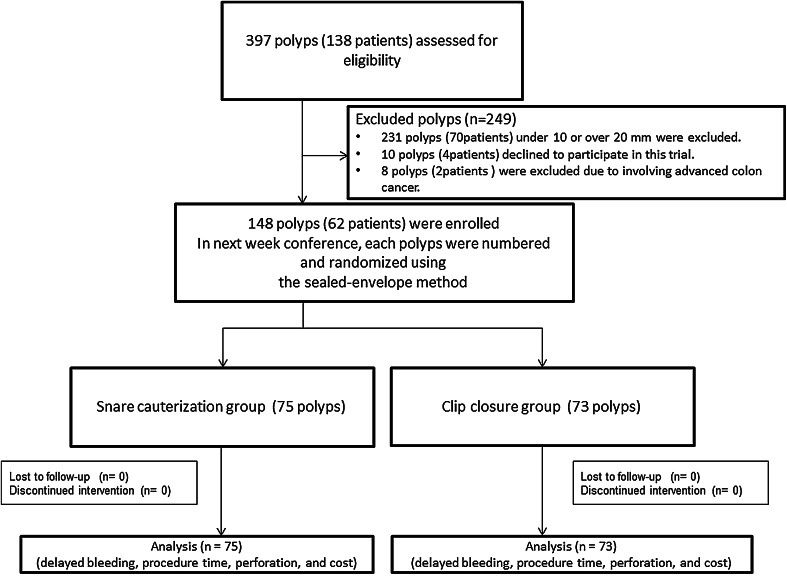
Flowchart of the prospective randomized trial

After discharge, we instructed the patients to rest for 10 days and to abstain from alcohol consumption and vigorous exercise (Fig. 1).

#### Snare cauterization procedure

**Fig. 2:**
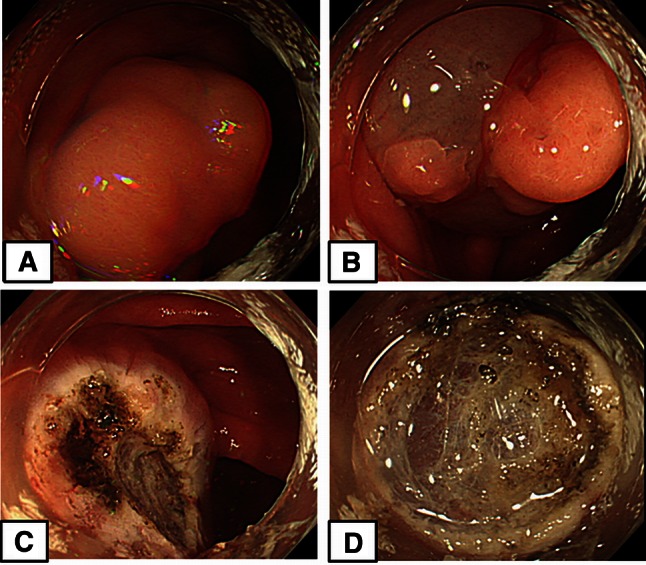
Snare cauterization procedure (Case 17). **A** A 14-mm-diameter polyp in the ascending colon. **B** In preparation for cauterization, sufficient saline was locally injected to create a protrusion >10 mm from the muscle layer. **C** Following snare resection, the snare tip was lightly pressed against the cut surface, and performed cauterization just above the muscle layer line. **D** We observed no blood vessels in the cauterized ulcer floor

We show one case of snare cauterization group for example. A polyp 14 mm in diameter was observed in the ascending colon (Fig. 2A). After this, sufficient saline was locally injected to create a protrusion >10 mm from the muscle layer (Fig. 2B). Subsequently, snare resection was performed, after which the snare tip was protruded by 2–3 mm only. Then, after sufficiently confirming the cut surface, we cauterized the surface starting from the mucous membrane at the edge (Fig. 2C). Using a tip hood, the stump was sufficiently observed from the front. If the presence of blood vessels was confirmed, the snare tip was lightly pressed horizontally approximately 20° against the cut surface. The surface was then cauterized while estimating the distance of the protrusion to the muscle layer line (Fig. 2D). The region was uniformly cauterized (video 1). Detailed observation revealed a sufficient saline protrusion remaining (Fig. 2C), and no blood vessels were observed in the cauterized ulcer floor (Fig. 2D).

**Fig. 3:**
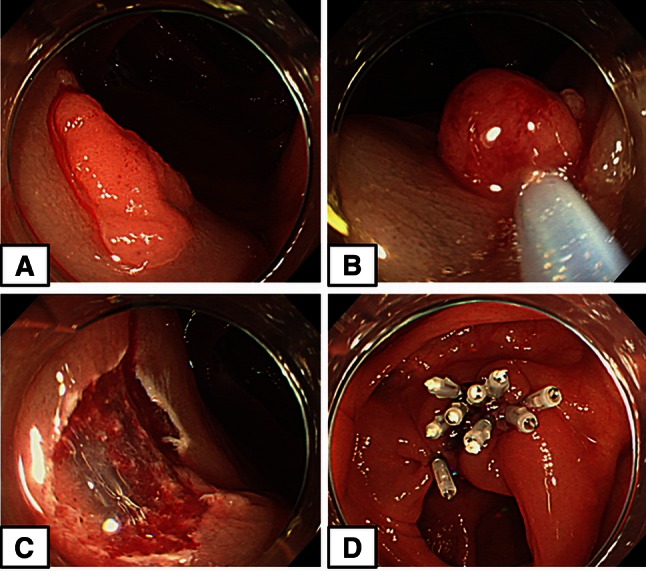
Clip closure group. **A** A 12-mm-diameter flat polyp in the ascending colon. **B** Resection of the polyp using a snare after local injection. **C** Blood vessels and bleeding on the cut surface. **D** Nine clips required to close the wound surface completely

In the clip group, the wound surface was closed starting from the edge of the cut surface, as per conventional procedure (Fig. 3A–D) (video 2).

#### Study sample size and enrollment

In our pilot study (five patients in the snare cauterization group, and five patients in clip closure group) (mean polyp size was 14.5 ± 2.69), we obtained the procedure times of wound closures (4.12 ± 1.24 min in the snare cauterization group vs. 11.6 ± 2.12 min in the clip closure group). Based on these results, we calculated the sample size for EMR of colorectal tumors under 20 mm in diameter by performing a statistical analysis using Graph Pad Prism 5 with the retrieved sample size (150 patients) at G* power using the effective size of 0.5 (http://biostat.mc.vanderbilt.edu/wiki/Main/PowerSampleSize).

#### EMR devices

- Endoscope: CF-HQ290 (Olympus Co., Tokyo, Japan)
- Snare: M00562321 (Boston Scientific Co., Tokyo, Japan)
- Clip: EZ CLIP (HX-610-135, Olympus Co., Tokyo, Japan) (Cost per clip: \975, US $9.56, €7.01)
- Injection needle: (NM-4U, Olympus Co., Tokyo, Japan)
- Local injection solution: Saline solution
- Incisional generator device: ERBE VIO300D (Elektromedizin, Tübingen, Germany; setting: Swift mode 45w, effect 4)

#### Outcomes

The primary assessment item was the wound surface treatment time (from immediately after polyp resection to wound surface treatment completion).

The secondary assessment items were as follows:

1. The incidence of delayed bleeding 1–7 days after EMR in the snare cauterization and clip closure groups.
2. Perforation incidence 1–7 days after EMR in the snare cauterization and clip closure groups.
3. Difference in medical costs between the snare cauterization and clip closure groups.

#### Ethical considerations

The ethics committees of Ehime Rosai Hospital (approval No. 43) and Kagawa University approved this study. The patients provided verbal and written informed consent.

#### Trial registration

University Hospital Medical Information Network (No. 000013473)

#### Statistical analysis

Data were analyzed between the groups using Mann–Whitney U test, Non-repeated measures ANOVA test, and the significance level was set at *P* < 0.05. Statistical analyses were performed using Graph Pad Prism version 5 for Windows (Graph Pad Software, San Diego, CA, USA).

## Results

In the snare cauterization group, polyps were detected at the ascending colon (10 lesions; 13 %), transverse colon (11 lesions; 15 %), descending colon (10 lesions; 12 %), sigmoid colon (24 lesions; 32 %), and rectum (21 lesions; 28 %). As for the macroscopic classification of the polyps, 0-Ip, Isp, Is, IIa, and IIc accounted for 7 (9 %), 17 (23 %), 23 (30 %), 25 (33 %), and 3 lesions (5 %), respectively. Meanwhile, the clip group, the locations where the polyps were detected were as follows: the ascending colon (9 lesions; 12 %), transverse colon (12 lesions; 16 %), descending colon (7 lesions; 10 %), sigmoid colon (25 lesions; 34 %), and rectum (20 lesions; 27 %). As for the macroscopic classification of the polyps, 0-Ip, Isp, Is, IIa, and IIc accounted for 6 (8 %), 18 (25 %), 21 (28 %), 26 (33 %), and 2 lesions (3 %), respectively. We observed no significant differences between the groups in terms of location, and macroscopic finding of the polyps.

Anticoagulants were used in 19 and 24 % of the polyps in the snare cauterization and clip closure groups, respectively. Antiplatelet drugs were used in 16 and 19 % of the polyps, respectively, without significant difference between the groups (*P* = 0.15 vs. 0.18). Among antiplatelet drugs, Aspirin/Ticlopidine Hydrochloride/Clopidogrel Sulfate were taken in 3/1/1 polyps, respectively, in the snare cauterization group, and 2/3/1 patients, respectively, in the clip group. There was no significant difference between the groups (*P* = 0.23) (Table 1).

**Table 1:** Baseline characteristics of the polyps in the two randomized groups

	Snare cauterization group (75 polyps)	Clip group (73 polyps)	*P* value^a^
Location of polyps			
Cecum-ascending	10 (13 %)	9 (12 %)	0.31
Transverse	11 (15 %)	12 (16 %)	0.4
Descending	10 (12 %)	7 (10 %)	0.32
Sigmoid	24 (32 %)	25 (34 %)	0.31
Rectum	21 (28 %)	20 (27 %)	0.32
Macroscopic classification of polyps			
Pedunculated (Ip)	7 (9 %)	6 (8 %)	0.52
Semipedunculated (Isp)	17 (23 %)	18 (25 %)	0.43
Sessile (Is)	23 (30 %)	21 (28 %)	0.46
Superficially elevated (IIa)	25 (33 %)	26 (36 %)	0.34
Superficially depressed (IIc)	3 (5 %)	2 (3 %)	0.54
Oral administration of anticoagulants, no. of polyps	6 (19 %)	7 (24 %)	0.15^a^
Oral administration of antiplatelets, no. of polyps	5 (16 %)	6 (19 %)	0.18^a^
(Aspirin/ticlopidine hydrochloride/clopidogrel sulfate), no. of polyps	3/1/1	2/3/1	0.23^b^

The data are presented as ‘n (%)’

^a^Mann–Whitney U test

^b^Non-repeated measures ANOVA test

Table 2 shows results for the two randomized groups. We observed no significant difference in pre-resection polyp size between the snare cauterization and clip closure groups (15.5 ± 2.60 mm vs. 15.3 ± 2.84 mm, respectively; *P* = 0.14). We also observed no significant difference in post-snare resection cut section size (18.5 ± 1.30 mm vs. 17.9 ± 2.21 mm, respectively; *P* = 0.20). In the snare cauterization group, the cautery diameter was 19.3 ± 1.10 mm. From polyp resection, the time required for wound surface treatment completion was 3.26 ± 1.57 min in the snare cauterization group and 12.7 ± 2.92 min in the clip closure group, thus demonstrating a significant difference (*P* = 0.0001). Although we observed delayed bleeding until day 7 after EMR in two polyps in the clip group, we observed no delayed bleeding in the snare cauterization group (*P* = 0.098). In the two cases of delayed bleeding, we observed that bleeding occurred between 2 and 7 days after EMR. We controlled the bleeding using hemostatic forceps and clips in two polyps, respectively, and we observed no subsequent bleeding. We did not observe perforation in either group. We used a mean number of 5.17 clips for post-EMR wound surface closure. In the histopathological examinations of the resected polyps, we observed no significant differences between the groups in the proportions of adenocarcinomas, tubular or tubulovillous adenomas, serrated adenomas, hyperplastic polyps, and others. We used 720 clips (738 after counting those that were not applied appropriately), which cost \523,410, US $5,136.50, or €3,765.5.

**Table 2:** Results for the two randomized groups

	Snare cauterization group (75 polyps)	Clip group (73 polyps)	*P* value^a^
Polyp size (mm), mean ± SD	15.5 ± 2.60	15.3 ± 2.84	0.14^a^
Size of cutting section (mm), mean ± SD	18.5 ± 1.30	17.9 ± 2.21	0.2^a^
Snare cautery diameter (mm), mean ± SD	19.3 ± 1.10	–	
No. of delayed bleeding cases (POD 1–7)	0 (0 %)	2 (2.7 %)	0.098^a^
No. of perforation cases (POD 1–7)	0 (0 %)	0 (0 %)	
Time from polyp resection to EMR completion (min), mean ± SD	3.26 ± 1.57	12.7 ± 2.92	0.0001^a^
No. of clips, mean	0	5.17	
Cost of all clips (¥/US $/€) (average exchange rate, July, 2014)	0	523,410/5136.5/3765.5	
Treatment measure for delayed bleeding			
Hemostatic forceps	0	1 (1.3 %)	
Clipping	0	1 (1.3 %)	
Histological type of polyps			
Adenocarcinoma	15 (20 %)	16 (22 %)	0.41^a^
Tubular or tubulovillous adenoma	56 (74 %)	52 (71 %)	0.39^a^
Serrated adenoma	2 (2 %)	2 (2 %)	0.81^a^
Hyperplastic polyp	1 (2 %)	1 (2 %)	0.81^a^
Others	1 (2 %)	2 (3 %)	0.79^a^

The data are presented as ‘n (%)’

^a^Mann–Whitney U test

## Discussion

As exposed blood vessels in the artificial ulcer floor after ESD have to be cauterized with hemostatic forceps, we were able to cauterize the wound surface uniformly in a much shorter time than required in clip closure using this snare cauterization technique as well. In this study, after performing snare resection of the wound surface after EMR, in which we created a protrusion with sufficient saline solution, subsequently, we were able to prevent the delayed bleeding only using the 2–3 mm snare tip just after sufficient observation of the cut surface, regardless of the presence of bleeding. We then cauterized the blood vessels in the submucosa from the protruded mucosa; we did so from a horizontal direction of approximately 20° against the cut surface of the protrusion. The procedure also does not require the application and removal of clips and prevents secondary mucosal bleeding caused by the clips. Accordingly, we did not observe delayed bleeding in any of our patients. This method is considered superior to clip closure in terms of procedure time, and the preventing effect of delayed bleeding for wound surface treatment after EMR between over 10-mm and under 20-mm polyps. With regard to resected tumor size, no consensus has yet been reached as to whether colorectal polyps <5 mm should be resected or whether endoscopic resection should be applicable for polyps that enlarge or undergo other morphological changes during follow-up. However, in 2012, Zauber et al. [4] reported that among colorectal polyps, all adenomatous polyps, regardless of size, yield a 53 % reduction in future mortality from colorectal cancer after resection. In addition, novel therapeutic techniques for lesions to which polypectomy or EMR was conventionally applied have been recently reported. In particular, increasing attention has been given to cold polypectomy for colorectal polyps <10 mm. After performing cold polypectomy for 1,015 lesions, Repici et al. [5] reported 2.2 % per-patient and 1.8 % per-polyp bleeding rates immediately after operation. Thirty days after operation, a favorable delayed bleeding incidence of 0 % was achieved, thus demonstrating the safety of the technique. The use of magnifying endoscopy with narrow-band imaging allows for easy differential diagnosis of neoplastic and non-neoplastic polyps [6–10]. Based on future adenoma-carcinoma sequencing, neoplastic polyps (including those <10 mm in diameter) have a high possibility of developing into cancer. Therefore, as diagnoses become more accurate, resection via cold polypectomy after diagnosis using magnifying endoscopy with narrow-band imaging may contribute to improved patient prognosis. In a prospective randomized trial in which cold polypectomy was performed without discontinuing anticoagulation therapy, the incidence of delayed bleeding was 0 % [11]. Thus, resection via proactive cold polypectomy could be recommended for neoplastic colorectal polyps <10 mm. Moreover, owing to the high tumor-bearing rate of lesions >20 mm in diameter, en bloc resection via ESD is desirable, as en bloc resection is difficult for such lesions [12, 13]. However, in the only report on wound surface treatment after EMR between over 10-mm and under 20-mm polyps, Liaquat et al. [3] retrospectively examined 524 lesions and reported the following: the incidence of delayed bleeding was 9.7 % without clip closure and was reduced to 1.8 % with clip closure. However, they also reported that prospective randomized trials are necessary. Therefore, to some extent, our randomized prospective study might provide one of post-EMR treatments for between over 10-mm and under 20-mm polyps. As for medical costs, one clip costs \975, US $9.56, or €7.01, with complete closure of a nearly 20-mm wound surface requiring an average of five clips. Performing cauterization with the same snare used for resection also just a little contributed to a medical cost reduction. In conclusion, in the treatment of wound surfaces after EMR between over 10-mm and under 20-mm polyps, this method is considered superior to clip closure in terms of procedure time, and not inferior to clip closure in terms of the preventing effect of delayed bleeding.

## Electronic supplementary material

Below is the link to the electronic supplementary material.

Supplementary material 1 (MPG 51774 kb)

Supplementary material 2 (MPG 16944 kb)

Supplementary material 3 (DOCX 13 kb)
