# Supplementary material for: Simple and reliable treatment for post-EMR artificial ulcer floor with snare cauterization for 10- to 20-mm colorectal polyps: a randomized prospective study (with video)
Source: Surg Endosc. 2014 Dec 6;29(9):2818–24. doi: 10.1007/s00464-014-3983-y (PMC4541705; doi:10.1007/s00464-014-3983-y)
Supplement: Supplementary file 3 — Supplementary material 3 (DOCX 13 kb) [file 464_2014_3983_MOESM3_ESM.docx]

**Video 1**

1. A 14-mm-diameter polyp in the ascending colon.

2. Sufficient saline solution locally injected to create a protrusion > 10 mm from the muscle layer.

3. After snare resection, the snare tip was protruded by 2–3 mm only.

4. Cauterization was performed horizontally at approximately 20° against the cut surface.

5. The distance of the protrusion to the muscle layer line was constantly estimated.

6. No blood vessels were observed in the cauterized ulcer floor.

**Video 2**

1. A polyp 15 mm in diameter was observed in the ascending colon.

2. After sufficient saline was locally injected, snare resection was performed.

3. 3 exposed vessels were seen in the ulcer floor after EMR.

4. We used Clips of which name was EZ CLIP produced by Olympus Co., Tokyo, Japan.

5. First clipping was placed at the right edge of the ulcer floor.

6. 9 clips were placed from right side to left side of the ulcer one after another.

7. It took about 2.5 min to place one clip.

8. Closure by 9 clips required around 23 min.

9. The clip closure group required longer procedure time to close the ulcer.
